# *Leishmania* cytochrome b gene sequence polymorphisms in southern Iran: relationships with different cutaneous clinical manifestations

**DOI:** 10.1186/s12879-018-3667-7

**Published:** 2019-01-29

**Authors:** Iraj Mohammadpour, Gholam Reza Hatam, Farhad Handjani, Farzaneh Bozorg-Ghalati, Daniel PourKamal, Mohammad Hossein Motazedian

**Affiliations:** 1 Department of Medical Parasitology and Mycology, School of Medicine, Shiraz University of Medical Sciences, Shiraz, Iran; 2 Molecular Dermatology Research Center, Department of Dermatology, School of Medicine, Shiraz University of Medical Sciences, Shiraz, Iran; 3 Department of Molecular Pathology, School of Medicine, Shiraz University of Medical Sciences, Shiraz, Iran; 4 Fajr Health Center, Shiraz University of Medical Sciences, Shiraz, Iran; 5 Basic Sciences in Infectious Diseases Research Center, School of Medicine, Shiraz University of Medical Sciences, Shiraz, Iran

**Keywords:** *Leishmania*, Minicircle kDNA, Cytochrome b (Cyt b), Sequencing, Phylogenetic analysis, Iran

## Abstract

**Background:**

Cutaneous leishmaniasis (CL) caused by *Leishmania* species, is a geographically extensive disease that infects humans and animals. CL is endemic in half of the 31 provinces of Iran, with 29,201 incidence cases reported in Fars province from 2010 to 2015. CL is polymorphic and may result in lesions characterized by different clinical features. Parasite genetic diversity is proposed to be one of the factors affecting the clinical outcome and lesion characteristics in CL patients. However, there is still very limited data regarding the genetic variation of *Leishmania* spp. based on the sequencing of Cytochrome b (Cyt b) gene.

**Methods:**

All patients originated from endemic regions in Fars province. The amplification of the Cyt b gene from isolates of 100 patients with disparate clinical forms of CL was accomplished using Nested-PCR. Sequence analysis of the amplified Cyt b was used to scrutinize the genetic variations among *Leishmania* isolates and connect the results with clinical pictures. The clinical demonstrations were basically of two types, typical and atypical lesions. Molecular phylogenetic tree was constructed using the Neighbor-Joining method, with species/strains from this study compared to species/strains from other geographical regions.

**Results:**

*Leishmania major* was identified as the predominant infecting *Leishmania* spp. (86% of cases), with the remainder of cases being infected by *Leishmania tropica.* Clinical examination of patients revealed 12 different clinical CL forms. Among *Leishmania* samples analyzed, five distinct haplotypes were recognized: three in *L. major* and two in *L. tropica*. We found a correlation between clinical outcomes and Cyt b sequence variation of *Leishmania* spp. involved. Moreover, we observed a higher presence of polymorphisms in *L. major* compared with *L. tropica*. This difference may be due to the different eco-epidemiologies of both species, with *L. tropica* being an anthroponosis compared to *L. major*, which is a zoonosis.

**Conclusions:**

The sequence analysis of Cyt b gene from 25 *L. major* and *L. tropica* strains demonstrated genetic variability of *L. major* and *L. tropica* causing CL in southern Iran, and a feasible connection amid the genetic heterogeneity of the parasite, geographical source and clinical appearance of the disease in human was detected.

## Background

Cutaneous leishmaniasis (CL), is a vector-borne zoonotic infectious disease caused by protozoan parasites of the genus *Leishmania* (Kinetoplastida, Trypanosomatidae) [1, 2]. It is transferred to humans through the bite of infected female phlebotomine sand flies of the genera *Phlebotomus* and *Lutzomyia* [3]. CL can cause by 21 *Leishmania* spp. and result in a wide spectrum of clinical manifestations in humans, with the infecting species being a great determinant of clinical outcome [4]. Contingent upon the species of *Leishmania* involved, humans and a large spectrum of mammals operate as reservoirs [5]. The disease is endemic in the tropical and subtropical regions of 98 countries across four continents. More than two thirds of new cases of CL transpire in six countries: Afghanistan, Algeria, Brazil, Colombia, Iran and Syria. An estimated of 0.7–1.3 million new cases occur worldwide annually [4, 6]. In Iran CL is caused by *Leishmania tropica*, (the agent for anthroponotic CL), *Leishmania major* (the agent of zoonotic CL), and rarely by *Leishmania infantum* [7–9]. In addition, it is common for different species to coexist in the same endemic areas, as seen in Fars province [7–9]. Single or multiple CL lesions typically occur on exposed parts of the body, such as face, and upper and lower extremities. Lesions usually self-heal in a few months, but may persist for many years (e.g. when super-infected or when located on joints), causing considerable morbidity and large scars [10].

There have been several reports from studies in Iran of atypical manifestations of the disease due to either uncommon sites of lesions or their unusual morphology. Lesions on atypical sites result in a more complex differential diagnosis [7, 8]. Uncommon clinical presentations include lupoid, verrucous, sporotrichoid, erysipeloid, eczematous, psoriasiform, zosteriform, keloidal, whitlow, paronychia, carcinoma-like, and midfacial destructive lesions [7, 8, 10–12]. Occasionally CL may manifest as isolated lymphadenopathy, or proceed into disseminated CL [13, 14].

The genetic heterogeneity may cause various phenotypes that manifest themselves in the variability of clinical features observed. Therefore, bestowed genetic variations in *Leishmania* populations, disease control and treatment could be challenging [15]. Multi-locus enzyme electrophoresis (MLEE) has traditionally been the gold standard for strain and species characterization [16, 17]. However, this customary classification has been challenged using nuclear and mitochondrial molecular markers, as they inclined to be more specific and stable [18]. Generally, DNA analysis demands reiterated copying of the genome, and the levels of inter- and intra-species diversity has to be taken into account. In order to appraise genetic characterization, a number of nuclear and extra nuclear DNA markers have been employed, including kDNA [19], GP63 [20], ITS1 [21], ITS2 [22], the N-acetylglucosamine-1-phosphate transferase gene [23], Cytochrome Oxidase II [24], Cytochrome b (Cyt b) [25], Miniexon [26], 7SL RNA [27], HSP70 [28], and Cysteine Proteinase B [29].

The mitochondrial genome has been disclosed to be a splendid origin of accessible genetic variation. Analysis of mitochondrial DNA has been used to understand the evolutionary biology at the inter- and intra-species levels [30, 31]. Mitochondrial DNA’s rapid rate of evolution, clonal patrimony, and absence of recombination makes it an ideal target for phylogenetic studies and a source of genetic markers of species and geographically confined populations [30, 31]. Mitochondrial kinetoplastid DNA (kDNA), arranged as mini and maxicircles, encodes proteins involved in energy production and ribosomal RNAs. Minicircles are about 800-bp in size, closely 600-bp variable and 200-bp conserved region, and repeated 10,000 times. Maxicircles are around 20–35 kb in size, and have 20–50 repetitions in the genome [30, 31]. The mitochondrial genome can encode gene products such as Cyt b in the cellular respiration cycle [32]. Cyt b is the principal redox catalytic subunit of Quinol, which is engaged in the electron transport process of the mitochondrial respiratory chain, and is regarded one of the most functional genes for phylogenetic studies [32–34].

In the present study, the sequence analysis of the amplified Cyt b gene was applied to investigate the presence of genetic polymorphisms among *Leishmania* isolates and correlate the findings with the clinical features of CL lesions in Fars province, Iran, over a 2-year period. Moreover, molecular phylogenetic relationships were assessed using Cyt b gene sequences obtained by this study and download from the GenBank database.

## Methods

### Ethics statement

The research protocol was endorsed (approval no. 94–7548) by the Institutional Ethics Clearance Committee (IECC) of Shiraz University of Medical Sciences and performed in accordance with international policies established by the Declaration of Helsinki.

### Written informed consent

Written informed consent (Code: IR.SUMS.REC.1394.S282) to participate in the study and use clinical images in publications was obtained from all adult patients and/or parents/legal guardians for children under the age of 16 years.

### Patients

One hundred patients who showed different types of CL lesions participated in this study. The patients were referred to the Dermatology Clinic of Saadi Hospital and Fajr Health Center from January 2015 to the end of December 2016. Selected patient lesions (the most recent, in case of multiple lesions) were first photographed and standard clinical descriptions for these lesions were obtained from the attending dermatologist. All patients originated from different rural and urban regions of Fars province. We excluded patients with clinical evidence of intercurrent bacterial or fungal superinfection of the ulcer, and those undergoing active treatment for CL. For each patient a structured questionnaire was completed with all demographic information about the patient (including code, age, sex, address, and travel history), the lesion (including the number of lesions, localizations, onset of the disease, and clinical characteristics), and therapeutic data. The questionnaire used in our study, was designed and developed for this study.

### Dermal scraping

For the margin dermal scraping, a deep disinfecting of the indurated active margin of the lesion with 70% ethanol was performed. Samples were taken by using a no. 15 disposable sterile surgical blade (Unicut, Chicago, IL, USA) to make an incision in the border of the lesion. Exudates and dermal tissues from the wall of the slit were scraped and smeared on two glass slides [7, 8]. The touch impression smears were air dried, methanol-fixed, stained with Giemsa (Merck, Darmstadt, Germany), and finally examined for amastigotes by microscopy.

### In vitro culture

Moreover, the dermal syringe-sucked fluid was collected under sterile conditions from each patient as follows: 0.1 mL of sterile saline solution was injected using an insulin syringe (1-mL, 25-gauge needle) into the nodule and the needle was rotated gently several times. A small amount of saline solution was injected into the tissue, and then aspirated. The fluid was transferred to two tubes of modified NNN culture medium. Modified NNN medium was biphasic, comprise of horse blood agar base and an overlay Locke’s solution [7, 8]. The specimens were inoculated into the medium and incubated at 25 °C. Every 2 to 3 days, the liquid phases of cultures examined under invert microscope, in order to observe motile promastigotes. Positive cultures were mass cultivated in RPMI-1640 medium (Gibco, Frankfurt, Germany) supplemented with 15% heat-inactivated Fetal Calf Serum (Gibco, Frankfurt, Germany), 2 mM L-glutamine, 100 U/mL Penicillin, and 100 μg/mL Streptomycin (Gibco, Frankfurt, Germany) [7, 8]. Nearly 2 × 10^6^ promastigotes were harvested by centrifugation (10,000 g for 10 min) and washed thrice in cold sterile PBS (pH 7.2). Parasites pellets were stored at − 20 °C until used.

### DNA extraction

Total genomic DNA was extracted from each clinical sample using the QIAamp^®^ DNA Mini Kit (QIAGEN, Hilden, Germany), according to manufacturer’s instructions. Following the centrifugation and washing steps, the DNA was eluted from the silica spin columns with 50-*μ*L elution buffer to increase its concentration. The quantity and quality of the extracted DNA was determined by measuring optical absorbance at 260 nm using a Nano spectrophotometer (NanoDrop^®^ 2000, Thermo Fisher Scientific, Wilmington, DE, USA). Each samples for PCR assays were prepared with aerosol-guard pipette tips to avoid contamination. All reactions were performed in appropriated places, following the good practice of laboratories to avoid sample contamination [7, 8]. The extracted DNA was stored at − 20 °C until used.

### kDNA semi-nested PCR

All samples (cultures and impression smears) were identified to *Leishmania* species level using kDNA primers before they were subjected to Cyt b amplification.

The conserved area of the minicircle kDNA from the *Leishmania* species of all the samples was amplified by semi-nested PCR using primers LINR4 (forward) (5′-GGG GTT GGT GTA AAA TAG GG-3′), LIN17 (reverse) (5′-TTT GAA CGG GAT TTC TG-3′), and LIN19 (reverse) (5’-CAG AAC GCC CCT ACC CG-3′) for species identification [7, 8, 35].

PCR was performed in a Bio-Rad MyCycler Thermocycler (Hyland Scientific, Stanwood, WA, USA). The PCR conditions were composed of pre-denaturation at 94 °C for 5 min, then 40 cycles of denaturation at 94 °C for 30 s, annealing at 52 °C (LINR4 and LIN17) or 58 °C (LINR4 and LIN19) for 45 s, and extension at 72 °C for 1 min, followed by final extension at 72 °C for 10 min. Amplicons were analyzed on 1.5% agarose gels (AddGene, Watertown, MA, USA) by electrophoresis at 90 V in 1 × TAE buffer (40 mM Tris-acetate and 1 mM EDTA, pH 8.3) and visualized by UV light (Uvitec, Cambridge, UK) after being stained with GelRed^®^ (Biotium, Hayward, CA, USA). Cross-contamination was monitored by negative controls for sample extraction and PCR solutions.

### Cyt b nested-PCR

Maxicircle Cyt b gene was amplified using nested-PCR. Nest 1 primers corresponded to COIIIF (5′ - GTT TAT ATT GAC ATT TTG TAG ATT - 3′) and MURF4R (5′ - CGA CGA ATC TCT CTC TCC CTT - 3′). Nest 2 primers matched to LCBF1 (5′ - GGT GTA GGT TTT AGT TTA GG - 3′) and LCBR2 (5′ - CTA CAA TAA ACA AAT CAT AAT ATA CAA TT - 3′) [34].

The partial region of the Cyt b gene was amplified with *Pfu* DNA Polymerase (Agilent Technologies, Santa Clara, CA, USA) under the following conditions: initial denaturation at 94 °C for 5 min, followed by 40 cycles, each consisting of 30 s at 94 °C, 45 s at 58 °C (COIIIF and MURF4R) or 50 °C (LCBF1 and LCBR2), 1 min at 72 °C, and a final extension at 72 °C for 10 min. Electrophoresis and visualizing were performed under the same conditions as described above.

Roche Molecular Diagnostics Laboratories (Roche, Penzberg, Germany) synthesized all primers.

Reference strains of *L. major* (MHOM/IR/54/LV39) and *L. tropica* (MHOM/IR/89/ARD-L2) were used as positive controls.

### Sequencing

The amplified DNA fragments of both kDNA and Cyt b genes were visualized on 1.5% agarose gels, parallel with standard DNA marker (Fermentas, Vilnius, Lithuania) to permit sizing. The PCR products were extracted from gel sections using the QIAquick^®^ Gel Extraction Kit (QIAGEN, Hilden, Germany).

Sequencing of 200 ng of the amplified kDNA gene products were accomplished by using the LINR4 and LIN19 primers. Direct sequencing was performed to bridge gaps in nucleotide sequences.

Sequencing of the amplified Cyt b gene products were executed by using Nest 2 primers (LCBF1, LCBR2) and two specific internal primers LCBF4 (5′ – TGT TAT TGA ATA TGA GGT AGT G - 3′) and LCBR4 (5′ – GAA CTC ATA AAA TAA TGT AAA CAA AA - 3′). DNA sequencing was carried out on an ABI PRISM^®^ 3730*xl* Genetic Analyzer (Applied Biosystems, Foster City, CA, USA) by the Sanger dideoxy chain termination method using the Big Dye^™^ Terminator Cycle Sequencing Ready Reaction Kit (Applied Biosystems, Foster City, CA, USA). Sequence accuracy was confirmed by sequencing both directions through the sequencing service of Roche Molecular Diagnostics (Roche, Mannheim, Germany). Special attention was paid to the double peaks and the accurate direction of the sequences was guaranteed. The variations between and within *Leishmania* species, and the number of different nucleotides in each sequence was determined.

### Phylogenetic analysis

The raw nucleotide sequences and chromatograms of both forward and reverse directions were viewed and analyzed using the Chromas (2.6.6) program. The nucleotide sequences were aligned and analyzed using the MUSCLE multiple sequence alignment program [36]. Consensus sequences were compared with homologous sequences in the GenBank database using the BLAST algorithm [37]. The sequences were assembled and edited with the BioEdit (7.2.6) to identify single nucleotide polymorphisms (SNPs) [38]. Multiple alignments were performed with data related to *Leishmania* species from Iran and other countries deposited in GenBank. The parasite species were confirmed based on the homology with kDNA and Cyt b genes sequences from *Leishmania* reference strains. A molecular phylogenetic tree was constructed by the Neighbor-Joining (NJ) method and genetic distances were calculated with Maximum Composite Likelihood model using MEGA-X [39]. The reliability of the NJ tree was assessed by the bootstrap method with 1000 replications. *Leishmania equatorensis* was treated as out-group in Cyt b phylogenetic analysis.

### Statistical analysis

The Fisher’s Exact Test was used for analyzing the relation between clinical features and *Leishmania* species involved. All statistical analyses were performed using SPSS (SPSS 24.0, Chicago, IL, USA). A *P*-value < 0.05 was considered statistically significant.

### Nucleotide sequence accession numbers

The partial sequences of the Cyt b gene obtained in this study were deposited in the GenBank database under accession numbers KX176846, KY290231, KY360312-KY360314.

## Results

### Clinical results

From clinical standpoint, 58 out of 100 patients surmised to have CL were male. The patients were part of an incongruous population in Fars province, Iran. Their ages sorted from 0.7 to 89 years. The period of the cutaneous lesion fluctuated between 2 weeks to 2 years.

The relative distribution of *Leishmania* species in Fars province was shown to be heterogeneous. The majority of CL was due to *L. major* (86% of all cases), with the remainder due to *L. tropica*. While *L. major* was isolated from patients originating throughout Fars province, *L. tropica* was exclusively isolated from patients originating from the city of Shiraz (14% of *L. tropica* isolates reported).

Lesions were categorized into three main typical and nine atypical forms according to the clinical features. Three main types categorized as follows: 1- Elevated erythematous lesions smaller than 0.5 cm in diameter were defined as papular. 2- Elevated deeply seated erythematous lesions larger than 0.5 cm were elucidated as nodular. 3- Erythematous elevated lesions larger than 1 cm in diameter with ulcer were illustrated as ulcerative plaque. The majority of the patients had lesions over exposed parts of the body, most commonly hands and arms, followed by legs, face, and trunk. Sixty-six patients had one lesion, 19 patients had two lesions, and 15 patients had three or more lesions. The clinical features of the patients are summarized in Table 1.

**Table 1:** Clinical presentations in patients with cutaneous leishmaniasis

Clinical Patterns	No.	Location	Sex	Duration
Nodular	21	upper and lower extremities, Lip, Trunk, Neck	M and F	2 weeks-7 months
Ulcerative Plaque	19	upper and lower extremities, Face	M and F	3 weeks-2 years
Hyperkeratotic	19	upper and lower extremities, Face, Neck, Nose	M and F	3 weeks-10 months
Erythematous	9	upper and lower extremities, Face	M and F	3 weeks-5 months
Eczematous	9	upper and lower extremities, Face, Neck	M and F	5 weeks-8 months
Volcanic	8	upper and lower extremities	M and F	5 weeks-7 months
Multi-Lesional	5	upper and lower extremities, Trunk	M and F	3–6 months
Verrucous	4	Foot, Forehead	F	2–6 months
Papular	2	Hand, Finger	M and F	4 months
Psoriasiform	2	Hand, Ear	M and F	3–5 months
Carcinoma-Like	1	Eyelid	F	5 months
Recidivans Type (Lupoid)	1	Wrist	F	15 months

**Fig. 1:**
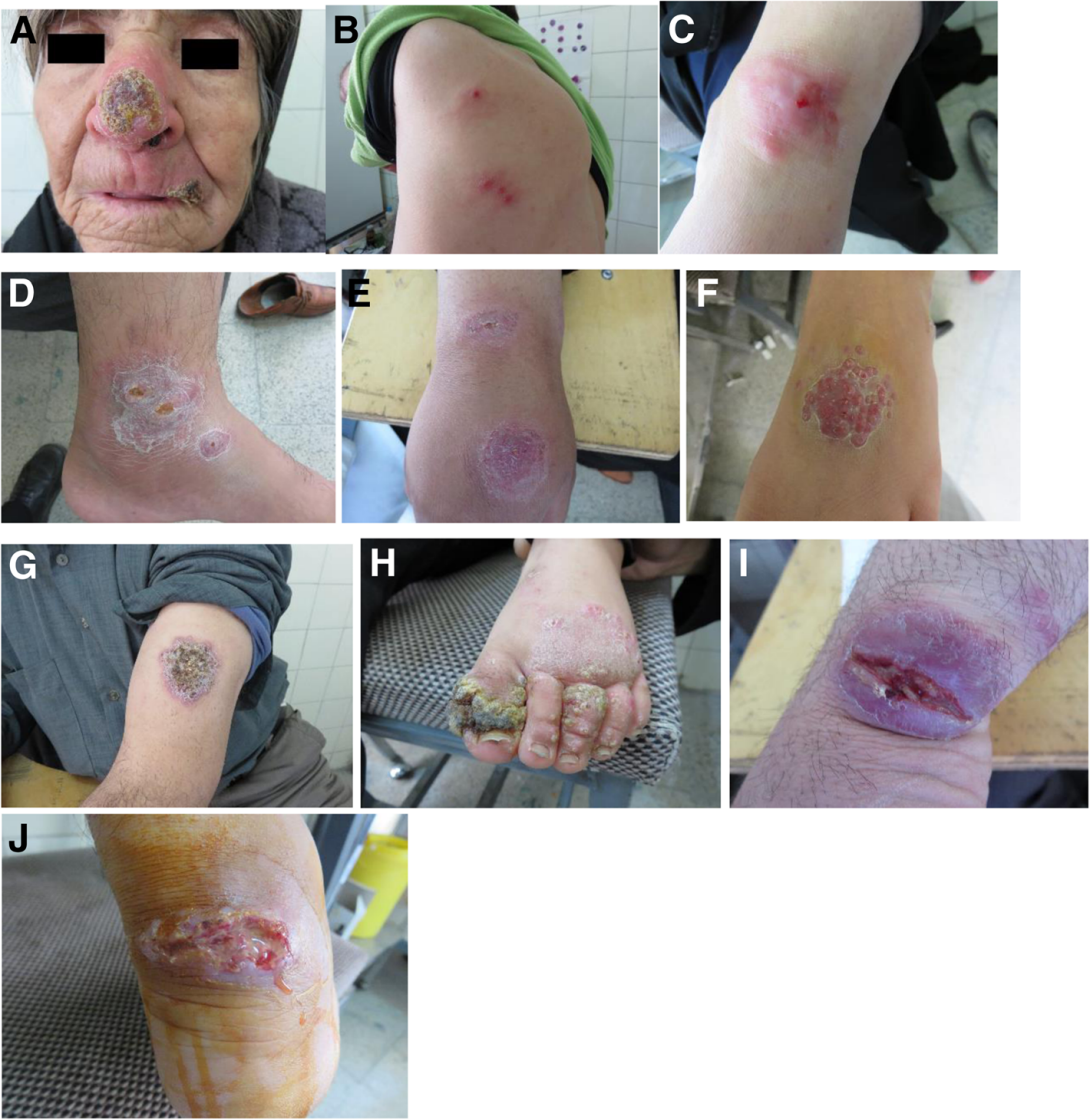
Clinical presentations of CL lesions caused by *L. tropica* and *L. major*: (**a**) Hyperkeratotic; (**b**) Multi-Lesional; (**c**) Erythematous; (**d**) Eczematous; (**e**) Psoriasiform; (**f**) Verrucous; (**g**) Discoid Lupus-Like; (**h**) Paronychia, Eczematous, and Hyperkeratotic; (**i**) Erythemato-Ulcerative; and (**j**) Wet Ulcerative Plaque

**Fig. 2:**
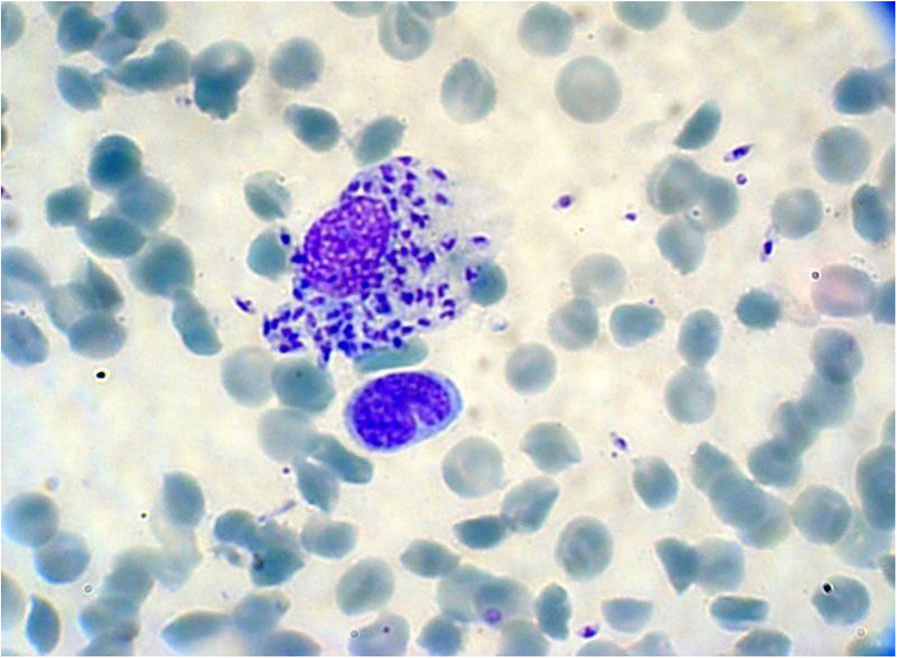
Touch impression smear. Numerous intracellular and scattered extracellular amastigotes are present (Giemsa stain; original magnification, × 1000). Kinetoplasts are visible in many amastigotes

The most common clinical presentation was nodular CL with 21 patients, followed by ulcerative plaque 19, hyperkeratotic 19, erythematous 9, eczematous 9, volcanic 8, multi-lesional 5, verrucous 4, psoriasiform 2, papular 2, carcinoma-like 1 [7, 8], and 1 recidivans-type (lupoid) (Fig. 1a-j). Numerous intracellular and scattered extracellular amastigotes were observed microscopically. Employing Giemsa, amastigotes are seen within the cytoplasm of macrophages as pale blue oval bodies with a dark blue nucleus and a small rod-shaped kinetoplast with a specified mitochondrial frame that contains extra-nuclear DNA (Fig. 2). CL was confirmed by microscopic examination of smears in 80% of 100 patients.

### Molecular, sequencing, and phylogenetic analysis findings

**Fig. 3:**
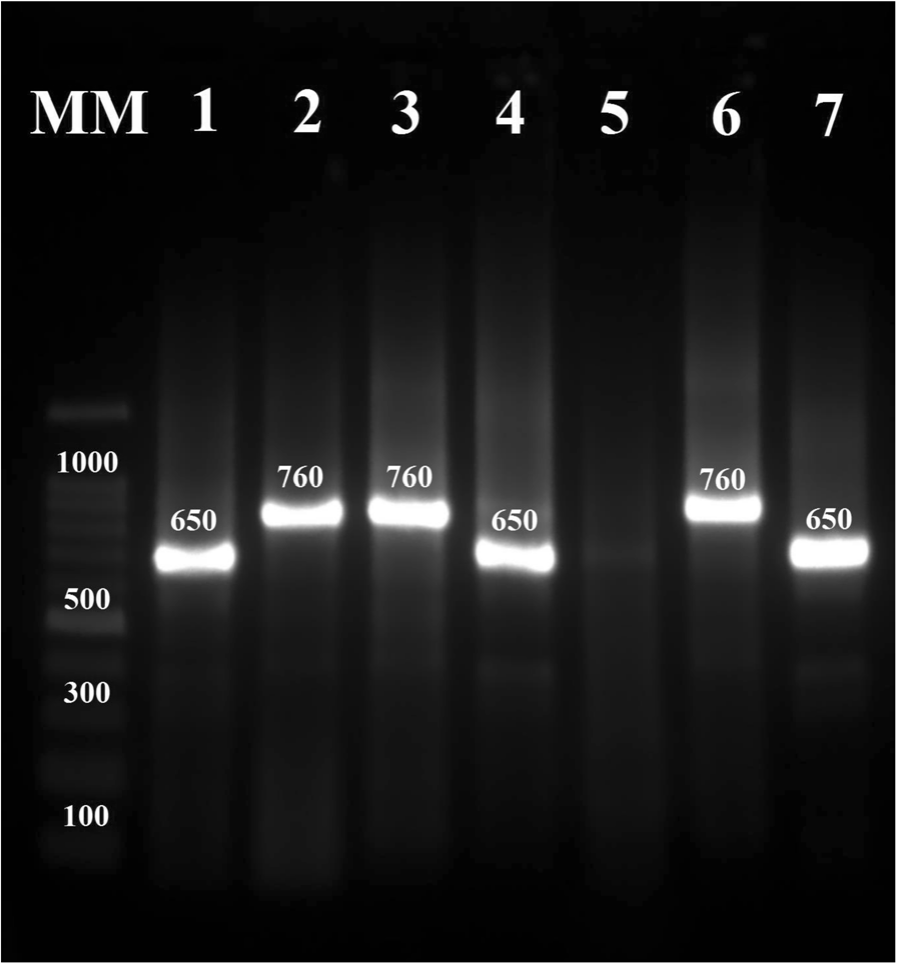
Electrophoresis of PCR products of DNA extracted from positive smears and cultures. The 8 lanes contained the products from positive controls of *L. tropica* (lane 6) and *L. major* (lane 7), negative control (lane 5), cutaneous lesions due to *L. major* and *L. tropica* (lanes 1–4), and a molecular marker (MM)

Semi-nested PCR was accomplished for amplification of the conserved area of the minicircle kDNA from the *Leishmania* spp. A 650-bp fragment was amplified for *L. major*, while a 760-bp fragment was amplified for *L. tropica* (Fig. 3). All 100 samples were sequenced for kDNA gene. The kDNA sequence analysis showed 14 cases of *L. tropica* and 86 cases of *L. major*.

**Fig. 4:**
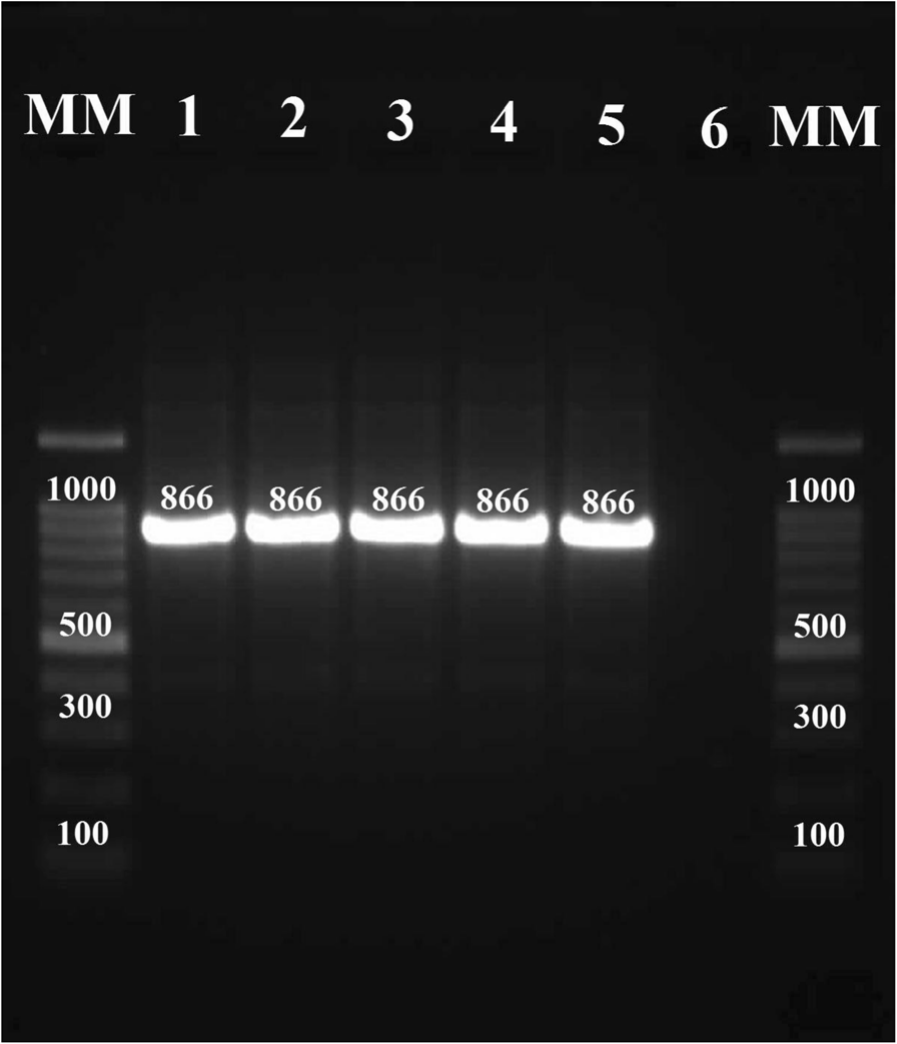
PCR result of *Leishmania* spp. isolated from CL lesions. The PCR product size is approximately 866 bp. The 8 lanes contained the products from positive controls of *L. tropica* (lane 4) and *L. major* (lane 5), negative control (lane 6), cutaneous lesions due to *L. major* and *L. tropica* (lanes 1–3), and molecular marker (MM)

Nested-PCR was executed for amplification of estimated size of 866-bp fragment of the internal Cyt b region in the second PCR reaction (Fig. 4). In this study, many of the 100 patients had similar skin lesions in size and clinical picture. Thus, 25 CL patients with various size lesions and different clinical outcomes were randomly selected and fully characterized for Cyt b gene sequencing. The resulting sequences of Cyt b gene were aligned and compared with those of existing sequences related to *Leishmania* in GenBank. The achieved sequences confirmed the presence of *L. major* and *L. tropica* that were recognized by the kDNA sequence analysis.

**Fig. 5:**
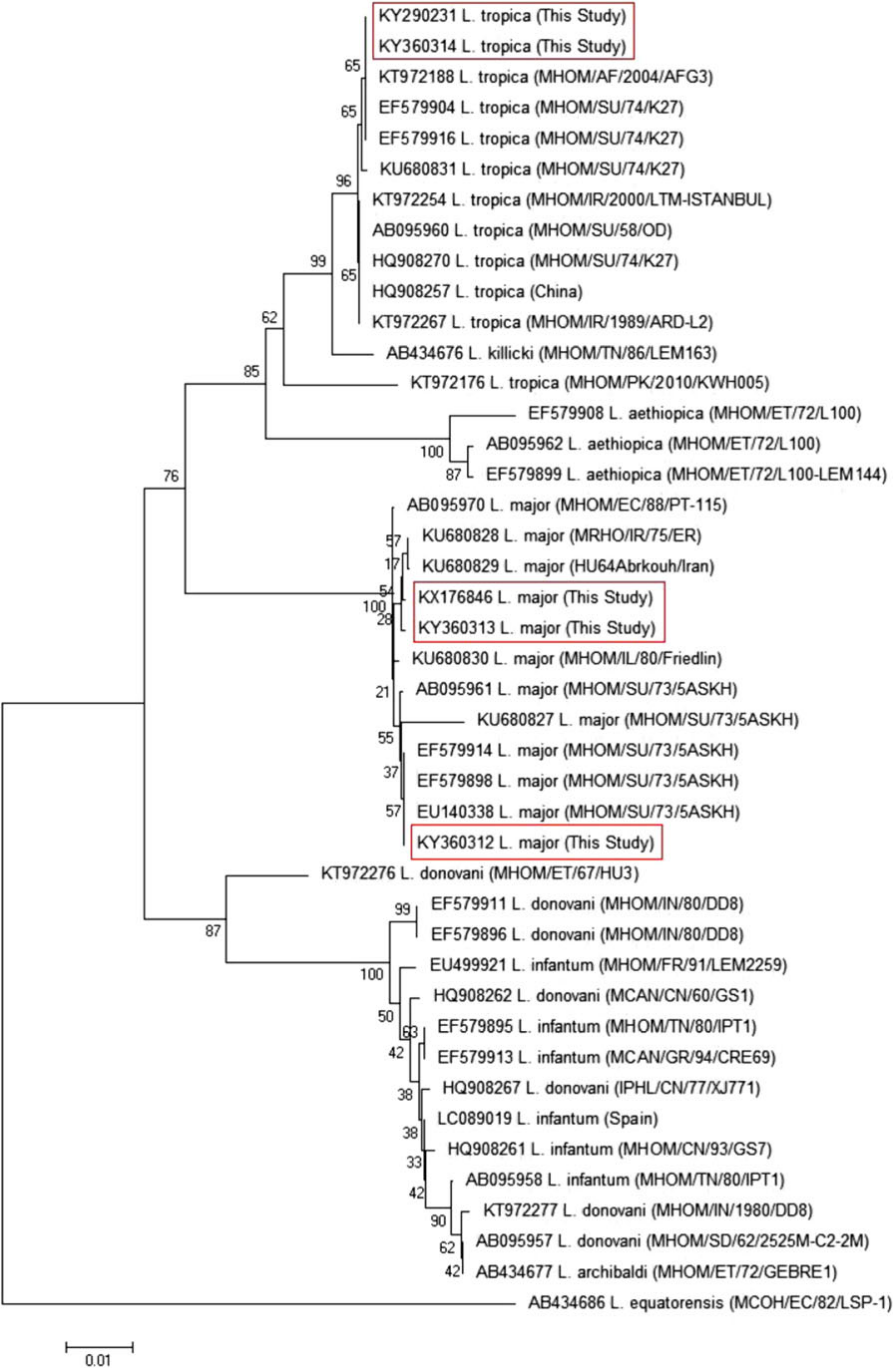
Molecular phylogenetic relationship among various *Leishmania* isolates to each other as inferred by Neighbor-Joining tree based on Cyt b gene. Numbers on branches are percentage bootstrap values of 1000 replicates. The evolutionary distances between sequences were computed using the Maximum Composite Likelihood method. The scale bar indicates an evolutionary distance of 0.01 nucleotides per position in the sequence. The reference sequences accession numbers are inserted. Evolutionary analyses were conducted in MEGA-X

In this study, two different strains of *L. major* (MRHO/IR/75/ER and MHOM/SU/73/5ASKH) and one strain of *L. tropica* (MHOM/SU/74/K27) were detected in Fars province, Iran. The Cyt b sequence analysis of 25 CL patients showed a 99–100% similarity to the previously published strains of *Leishmania* spp. The sequences of *L. major* patients with accession number KX176846 showed 99% identity to the published strains MRHO/IR/75/ER, and HU64, Abrkouh/Iran (KU680828 and KU680829). The sequences of *L. major* patients with accession number KY360312 showed 100% identity to the published strain MHOM/SU/73/5ASKH (EU140338, EF579898, and AB095961). Also, these isolates showed 99% identity to the published KU680827. The sequences of *L. major* patients with accession number KY360313 showed 100% identity to the published strain MRHO/IR/75/ER (KU680828). Also, these isolates showed 99% identity to the published strain HU64, Abrkouh/Iran (KU680829). The sequences of *L. tropica* with accession number KY360314 showed 100% identity to the published strain MHOM/SU/74/K27 (KU680831, HQ908270, and EF579904). Furthermore, the sequences of *L. tropica* with accession number KY290231 showed 99% identity to the published strain MHOM/SU/74/K27 (EF579904, HQ908270, and KU680831) (Fig. 5).

The common term of haplotype is a specific group of mutations or a collection of SNPs in the orthologous gene of the parasite. In this study, the haplotype diversity of Cyt b gene was observed to be higher in *L. major* population. Three haplotypes of Cyt b polymorphism of *L. major* were identified. In the sequencing result of *L. major* haplotype II (KX176846), thymine (T) is replaced by cytosine (C) at nucleotide position 258, C is replaced by T at nucleotide positions 394 and 801, and T is replaced by adenine (A) at nucleotide position 813. Alignment of the amino acid sequence corresponding to the non-edited region of haplotype II revealed Phe → Tyr substitution. Haplotype II was observed in the psoriasiform, and eczematous lesions.

In *L. major* haplotype III (KY360313), T is replaced by C at nucleotide position 280, C is replaced by T at nucleotide position 416, and T is replaced by A at nucleotide position 839. Alignment of the amino acid sequence corresponding to the non-edited region of haplotype III revealed two amino acid substitutions: One Trp → Arg substitution, and one Thr → Ile substitution. Haplotype III was principally observed in the carcinoma-like lesions.

Moreover, in *L. tropica* (KY290231), T is replaced by G at nucleotide position 810, and conversely at nucleotide position 811. Alignment of the amino acid sequence corresponding to the non-edited region of *L. tropica* (KY290231) revealed Leu → Cys substitution. This haplotype was essentially observed in the LR (lupoid) lesions. All variations occurred in microsatellite regions and were due to SNPs. In Fig. 5, the partial sequences of five haplotypes obtained in this study and deposited in the GenBank database, were analyzed.

**Fig. 6:**
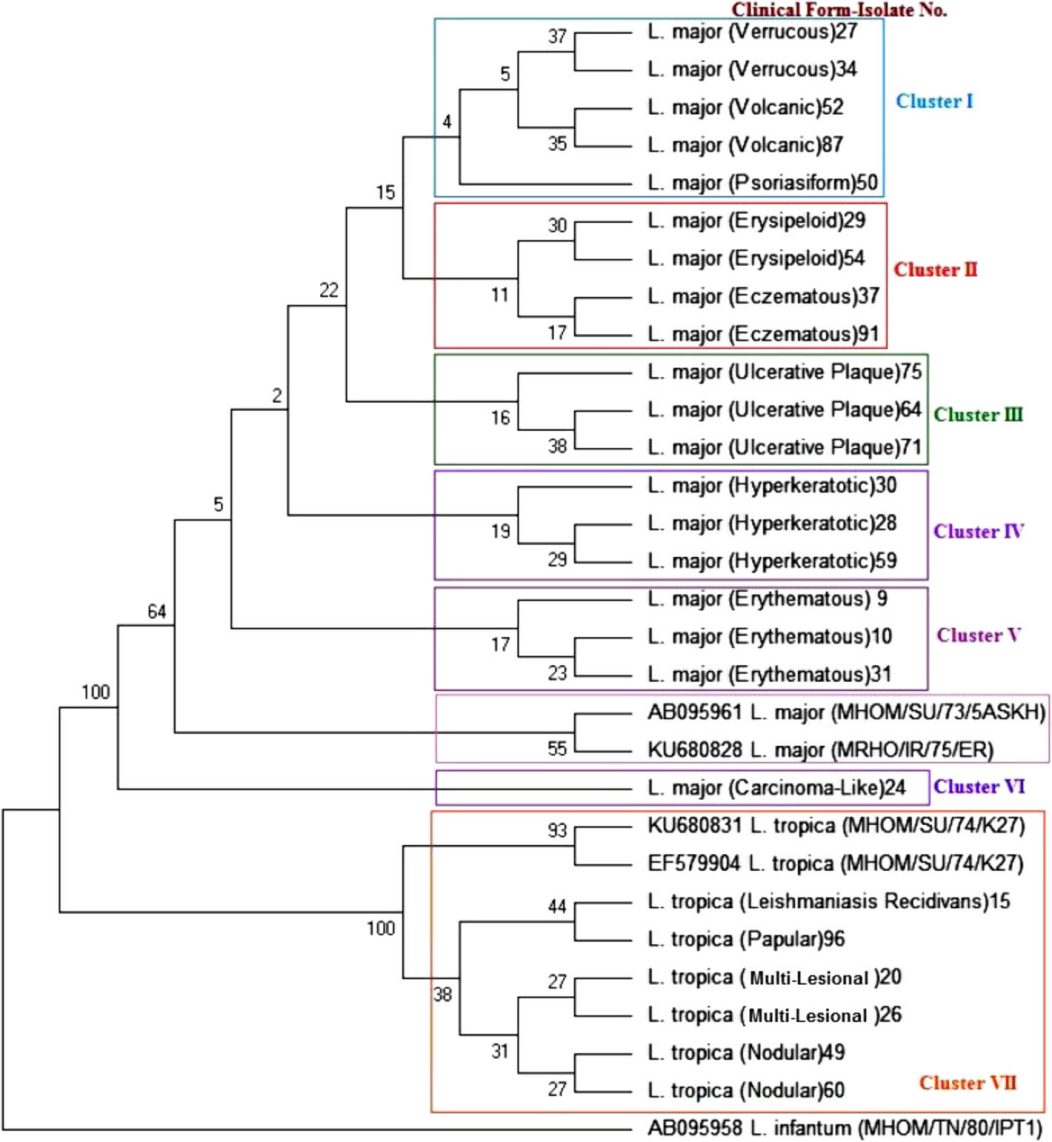
A phylogenetic consensus tree between all *Leishmania* isolates using MEGA-X and clustering algorithms. *L. infantum* (AB095958), two *L. major* (AB095961, KU680828), and two *L. tropica* (EF579904, KU680831) were used as out-group and standard isolates, respectively

The tree based on the classification of lesions grouped the 25 genotypes into 7 clusters (Fig. 6). Cluster I contained isolated strains from the verrucous, volcanic, and psoriasiform variants of CL patients who came from the same geographical region (isolates 27, 34, 50, 52, and 87). Cluster II included isolated strains from the erysipeloid and eczematous variants of CL patients who came from the same geographical origin (isolates 29, 37, 54, and 91). Cluster III comprised isolated strain from the ulcerative plaque of CL patients who came from the same geographical source (isolates 64, 71, and 75). Cluster IV embraced isolated strains from the hyperkeratotic variants of CL patients who came from the same geographical region (isolates 28, 30, and 59). Cluster V combined isolated strains from the erythematous variant of CL patients who came from the same geographical zone (isolates 9, 10, and 31). Cluster VI incorporated isolated strain from the carcinoma-like of CL (isolate 24). Withal, Cluster VII hugged isolated strains from the LR, papular, nodular, and multi-lesional variants of CL patients who came from the same geographical area (isolates 15, 20, 26, 49, 60, and 96). In Fig. 6, raw sequencing data of 25 Cyt b sequenced CL patients with different clinical pictures and sizes were used for phylogenetic consensus tree. The data shown in this phylogenetic consensus tree disclosed that those patients, who had the same clinical outcomes and came from the same geographical source, were infected with closely related strains of *L. major* in the phylogeny.

The analysis of the phylogenetic tree revealed two distinct clades: *L. major* and *L. tropica*. Within the clades intra-species divergence was more pronounced in *L. major* than in *L. tropica*. The Iranian strains of *L. major* and *L. tropica* found in this study were more similar to strains from the eastern and northern neighbor countries of Iran (Fig. 5).

## Discussion

Cutaneous leishmaniasis is a polymorphic disease that can divulge distinctive clinical outcomes, and is characterized by skin lesions and ulcers on exposed parts of the body, departing perpetual scars. CL is allotted in greater than half of the 31 provinces of Iran, with 29,201 incidence cases reported in Fars province from 2010 to 2015 [40]. Fars province in southern Iran is a hyper-endemic region of CL [41]. Early identification and genetic characterization of causative agents of CL using Cyt b gene or other genetic markers has been avail for appraisal of *Leishmania* polymorphisms, since infected *Leishmania* species are confederated with the clinical presentation and drug susceptibility.

In this study, we used Cyt b gene sequencing to study genetic diversity among 100 *Leishmania* isolates from the different parts of Fars province, Iran and to correlate the genetic polymorphism of the parasite with the clinical manifestations of the disease in humans. One of the advantages about using gene sequencing is the understanding of the inter- and intra-species genetic diversity of *Leishmania*. Cyt b is situated in the maxicircle part of the kinetoplast that is about 50 copies. There is sufficient degree of nucleotide sequence change amid *Leishmania* spp. genomes for characterization and heterogeneity aims [34]. Recently sequencing of the Cyt b gene has been employed with prosperity for *Leishmania* sp. identification [33, 42–47] and polymorphism [25, 34, 42, 43, 48–50]. Despite the low inter-species heterogeneity of the Cyt b gene, the key nucleotide positions depicted previously corroborate the potential of this gene as a molecular marker for *Leishmania* species characterization, not only in geographically related isolates, but also in widely separated regions [45].

The data from this study revealed genetic diversity of the Cyt b gene of *Leishmania* spp. isolated from a wide spectrum of clinical forms of CL in Fars province, Iran. This is in accordance with prior studies. Myint et al. [49] found three types of Cyt b polymorphism of *L. major* and no connection between clinical presentation and causal *Leishmania* parasites. Ramirez et al. [51] reported a high genetic diversity displayed by *L. panamensis* and *L. braziliensis* using Cyt b barcoding.

The genetic diversity of *Leishmania* spp. seen in academic research studies is dependent on a number of factors ranging from the parasite’s different eco-epidemiologies (e.g. are parasites isolated from humans, reservoir hosts or vectors; are they transmitted anthroponotically or zoonotically) to laboratory tools and molecular tools used (e.g. nuclear in contrast with mitochondrial DNA) [43]. Additionally, the occurrence of clonal reproduction and hybridization causes intrinsic genetic diversity in *Leishmania* [52, 53]. Of all these factors, sexual reproduction is the basic biological process that influences the population’s genetic structure. Many authors have reported evidence of hybrid formation and fortuitous bouts of genetic exchange or hybridization in *Leishmania* [54–57]. Clearly, infrequent or rare sessions of sexual recombination in normally asexual parasites can have a deep effect on the range of genetic diversity. It has been informed that increased transmission potential and a new form of CL is the result of hybrid formation between *L. major* and *L. infantum* [56, 57].

A high degree of genetic polymorphisms in *Leishmania* parasites based on ITS1 and kDNA genes has been reported previously in Iran [58–62], and in the neighboring country of Afghanistan [63, 64]. In a preceding study by Baghaei, mutual connection between the genetic heterogeneity of *L. major* and clinical presentations of ZCL in Isfahan, Iran based on PCR-RFLP of ITS gene in the ribosomal operon, has been investigated [58]. His study revealed that *L. major* is genetically highly polymorphic and a correlation may exist between genetic heterogeneity of the parasite and the clinical picture of the disease in human. The PCR-RFLP of the RNA polymerase II largest subunit (RPOIILS) gene of *L. major* has divulged genetic diversity in Iran [65]. The genetic variability of *L. major* from Iranian isolates have been disclosed antecedently by Single-Strand Conformation Polymorphism PCR (SSCP-PCR) and sequence analysis of the ITS gene [60]. The Permissively Primed Intergenic Polymorphic-PCR (PPIP-PCR) displayed further genetic heterogeneity amid the clinical isolates of *L. major* causing CL in Isfahan, Iran [66]. Supplementally, the genetic polymorphism of the rDNA gene of *L. major* has been informed in Fars province, Iran [67].

In addition, substantial heterogeneity has been studied and reported within the ITS gene of strains of *L. tropica* [59, 64, 68, 69]. Oryan et al. [61] and Shirian et al. [62] assessed the heterogeneity of *L. major* causing CL based on sequencing of kDNA and showed a high genetic diversity of the parasite and correlations among the geographical origin and the clinical outcomes of the disease. Moreover, conspicuous genetic variability has been exhibited within the *Nagt* gene amidst *L. tropica*, *L. major*, and *L. infantum* strains [70, 71], and by RAPD-PCR among *L. major* and *L. infantum* strains [72–74]. Considerable genetic diversity was detected among *L. major* strains from different endemic areas and even between some isolates of the same endemic area in Iran using the RAPD technique [73]. The latter result might be elucidated by substantial “Gene Flow” among isolates belonging to the same area [75].

The findings of higher molecular diversity in *L. major* isolated from tropical and subtropical regions of the Fars province in this study rather than *L. tropica* from the Shiraz region could be related to the greater number of animal reservoirs and diversity of sand fly fauna encountered in these regions [3, 5, 41].

In this study, an intelligible correlation was discerned between the Cyt b gene sequence polymorphism of isolates and clinical pictures of skin lesions. This is in conformity with previous studies [42, 49, 50]. Our results disclosed noteworthy variations in the clinical features of the CL caused by *L. major* secluded from different geographical regions of Fars province, Iran. The CL typically demonstrates as papules, scaled-crusted nodules, and ulcerative plaques. However, it may sometimes pose in various atypical clinical outcomes such as sporotrichoid, erysipeloid, lupoid, keloidal, eczematous, erythematous, psoriasiform, zosteriform, chancriform, hyperkeratotic, verrucous, whitlow, paronychia, carcinoma-like and other atypical exhibitions [7, 8, 10, 11]. Coherent with these data, in a prior study, assessment of four *L. major* isolates collected from four different endemic areas in Iran displayed diverse clinical and immunological patterns in BALB/c mice [76]. The different clinical expressions of CL depend on both intra-species genetic diversity of *Leishmania* and host immune status. Compound lesions have been portrayed in connection to *L. mexicana*, *L. braziliensis*, *L. tropica* and *L. major*, the mentioned last leading to primarily dermotropic types. In similar circumstances, the disease disseminates from the initial lesion by way of the lymphatic vessels, presenting subcutaneous nodules or localized adenopathy that have a similar appearance to lymphocutaneous sporotrichosis [10, 11].

In addition to the intra-species genetic variability of the *Leishmania*, host immune reaction performs a significant function in the clinical presentation of CL. For example, patients with defect of the T cell reply frequently improve an anergic condition named diffuse CL characterized by multiple nodular lesions full of amastigotes. Moreover, host genetic inheritance and bacterial habitat are contributed to the outcome of CL [77–80]. It has turned into limpid that the outcome of CL arises from an equilibrium between pro- and anti-inflammatory agents [81]. In CL patients, pathophysiology of disease is allied with a strong Th1 immune response to *Leishmania* antigens. Lesion dimension clearly connects with the immensity of *Leishmania* antigen- aroused TNF yield by peripheral blood mononuclear cells, and with the amount of flow TNF and IFN-γ producing CD4^+^ lymphocytes [82, 83]. Furthermore, there is an alliance between the strength of the inflammation and the frequency of CD8^+^ T cells exuding granzyme A [84].

Extra agents that have been asserted to affect the clinical outcome of CL comprise the place of inoculation, the total amount of the inoculated promastigotes, hormonal secretion quality, the quantity, quality and variety of food intake of the host, and the temperament of the final non-blood repast of the vector. Besides, agents like a non-native person, aged people communally, utilize of oral steroid drugs, immunodeficiency illnesses, and still lesion pollution with inorganic particles are able to modify the signs and symptoms of CL [85].

With relation to the effective causes of CL in Iran, the high usually recognized parasites were *L. major* and *L. tropica*, respectively. Dependent upon the results of this study, *L. major* is the supreme species liable for CL in this district. Three *Leishmania* spp. comprise of *L. major*, *L. tropica*, and sometimes *L. infantum* had been recognized as the causative agents of CL and ML collaborated with disparate clinical pictures in this territory [7–9]. The data procured in this study disclosed that those patients who had the similar clinical outcomes and came from the same geographical source were affected with almost linked strains of *L. major* in the phylogeny. Certain patients with various clinical configurations were situated in the equal bunch.

The established data from this study revealed that a correlation might be exist between the genetic variability of the parasite, clinical manifestation, and geographical source of the disease in humans. This is in agreement with previous studies [48–50].

## Conclusions

The sequence analysis of the *Leishmania* Cyt b gene showed genetic polymorphisms in *L. major* and *L. tropica* and a feasible correlation among the genetic heterogeneity of the parasite, geographical source and clinical outcome of the disease in human was found. Furthermore, these data confirm and emphasize the usefulness of Cyt b gene sequencing on *Leishmania* spp. genetic polymorphism and phylogenetic relationship analysis. Based on our findings, we believe that different clones of parasites or mixed populations are circulating in endemic regions of Fars province, Iran. It is significant to contemplate that the clinical configuration of CL does not solely be contingent upon the *Leishmania* species involved. Meanwhile, even though certain lesion characteristics maybe more commonly associated with a particular species, one should not rely on clinical patterns to anticipate any species involvement.

## Abbreviations

7SL: 7 Splicing Leader
A: Adenine
Arg: Arginine
C: Cytosine
CL: Cutaneous leishmaniasis
Cys: Cysteine
Cyt b: Cytochrome b
FCS: Fetal Calf Serum
G: Guanine
GP63: Glycoprotein 63
HSP: Heat Shock Protein
IFN-γ: Interferon γ
Ile: Isoleucine
ITS: Internal Transcribed Spacer
kDNA: kinetoplast DNA
Leu: Leucine
MLEE: Multi-locus enzyme electrophoresis
NJ: Neighbor-Joining
PBS: Phosphate Buffered Saline
Pfu: *Pyrococcus furiosus*
Phe: Phenylalanine
SNP: Single Nucleotide Polymorphism
T: Thymine
Thr: Threonine
TNF: Tumor Necrosis Factor
Trp: Tryptophan
Tyr: Tyrosine
